# Overexpression of *Map3k7* activates sinoatrial node-like differentiation in mouse ES-derived cardiomyocytes

**DOI:** 10.1371/journal.pone.0189818

**Published:** 2017-12-27

**Authors:** Kemar Brown, Stephanie Legros, Francis A. Ortega, Yunkai Dai, Michael Xavier Doss, David J. Christini, Richard B. Robinson, Ann C. Foley

**Affiliations:** 1 Greenberg Division of Cardiology, Weill Medical College of Cornell University, New York, New York, United States of America; 2 Department of Bioengineering, Clemson University, Charleston, SC, United States of America; 3 Department of Pharmacology, Columbia University Medical Center, New York, NY, United States of America; University of Kansas Medical Center, UNITED STATES

## Abstract

*In vivo*, cardiomyocytes comprise a heterogeneous population of contractile cells defined by unique electrophysiologies, molecular markers and morphologies. The mechanisms directing myocardial cells to specific sub-lineages remain poorly understood. Here we report that overexpression of TGFβ-Activated Kinase (TAK1/*Map3k7*) in mouse embryonic stem (ES) cells faithfully directs myocardial differentiation of embryoid body (EB)-derived cardiac cells toward the sinoatrial node (SAN) lineage. Most cardiac cells in *Map3k7*-overexpressing EBs adopt markers, cellular morphologies, and electrophysiological behaviors characteristic of the SAN. These data, in addition to the fact that *Map3k7* is upregulated in the sinus venous—the source of cells for the SAN—suggest that *Map3k7* may be an endogenous regulator of the SAN fate.

## Introduction

In mammalian embryos, the SAN is located in the right atrial wall of the heart between the superior vena cava and the crista terminalis [1] and acts as the site of impulse initiation in the heart. It also serves as the heart’s pacemaker by adjusting the rate of beating in response to environmental cues. SAN cells are molecularly, morphologically, and electrophysiologically distinct from both atrial and ventricular myocardial cells.

Identification of SAN cells ex-vivo is complicated by a number of factors, including the overall heterogeneity of the SAN (reviewed in Barbuti and Robinson [2]). Nevertheless, a number of criteria have become widely accepted for this purpose. Characteristics that are considered to be essential benchmarks for SAN cells include rapid beat rate, automaticity, action potential morphologies that include diastolic depolarization, the ability to speed up and slow down beating in response to small molecules that impact the concentration of cyclic AMP, and functional expression of the inward funny channel (I_f_) [3]. The I_f_ channel belongs to a family of *Hyperpolarization-Activated Cyclic Nucleotide-Gated Potassium Channel 4* (*Hcn4*) proteins [4, 5], of which *Hcn4* is the dominant member during mouse SAN development [6].

SAN cells can also be distinguished from working myocardial cells by their expression of particular molecular markers and the activation of a SAN-specific transcriptional program. Genetic studies in mouse embryos have begun to define the transcriptional network that mediates SAN differentiation. *Tbx5* expression within the heart tube establishes expression of *Shox2*, which both inhibits expression of *Nkx2*.*5* and activates expression of the transcriptional repressor, *Tbx3* [7, 8]. *Tbx3*, in turn, inhibits atrial chamber specification and activates SAN-specific gene expression, including *Hcn2 and Hcn4*, among other factors [9–11] (reviewed in [12]). Meanwhile, high levels of *Nkx2*.*5* activity in the working myocardium repress the aforementioned transcriptional network, resulting in a progressive, regional refinement of SAN-specific gene expression to the node and sinus horns [13]. Another characteristic of SAN cells is a low abundance of the inward rectifier current [14] but relatively high levels of the L-type calcium channel *Cav1*.*3* [15, 16].

Cardiomyocytes derived from mouse or human ES cells or from induced pluripotent stem cells (IPSCs) comprise fewer than 20% nodal-like cells [11, 17–19]. However, several approaches have been developed in recent years to isolate cell populations that are highly enriched for SAN-like cells. For example, both the addition of suramin and the inhibition of *Neuregulin/ErbB* signaling [11, 19] appear to preferentially expand the SAN lineage over other cardiac lineages. Highly purified populations of SAN cells can be isolated based on the expression of SAN-specific markers. This can be done by using either genetically engineered tags [20], or the cell surface marker CD166, which serves as a specific marker of SAN progenitors during early development [16]. There are also promising early results using transcription factor overexpression to achieve directed differentiation of SAN lineages. Recently, Kapoor et al. demonstrated that genetic transduction of *Tbx18* into neonatal rat ventricular myocytes (NRVMs) could convert them into pacemaker-like cells [21], suggesting that transcription factor regulation might be sufficient to drive cells to the SAN fate. Since then, overexpression of *Tbx3* [22], *Shox2* [23] and *Islet-1 (Isl1)* [24] have all been demonstrated to activate SAN-like characteristics in cardiomyocytes derived from pluripotent stem cells. Altogether these data suggest that transcription factors can activate or selectively direct the differentiation of SAN cells in populations of differentiating EBs. Finally, Protze et al. demonstrated that it was possible to isolate a population of cells that were highly enriched for the SAN fate without the addition of transgenes [25]. However, full realization of this potential will require a more thorough understanding of the signaling pathways that lead cells to adopt the SAN fate during development.

We recently identified the *TGFβ-Activated Kinase* (*TAK1*/ *Map3k7*) signaling pathway [26] as a potential mediator of cardiac differentiation [27, 28]. Previous studies have shown that *Map3k7* is required for cardiac differentiation of P19 cells [29] and that mice possessing homozygous deletions of *Map3k7* have cardiac defects [30]. In addition, mice that express a dominant interfering form of *Map3k7* in the heart die shortly after birth due to conduction abnormalities [31]. Together these findings suggest that *Map3k7* may play a specific role in the differentiation of the cardiac conduction system.

To examine this possibility, we produced ES cell lines overexpressing *Map3k7* and found that nearly all of the cardiac cells that differentiated from these cell lines had gene expression and electrophysiological characteristics of SAN cells, including expression of the I_f_ channel. Differentiated cells also showed decreased transcriptional expression of markers for the working myocardium such as *Mhcα*, *Mhcβ* and *Mlc2a*, in combination with a near total absence of *Cx43*; they also upregulated the the transcriptional network that directs endogenous SAN differentiation. Finally, we observed changes in the expression of SAN related genes as early as day 5 after EB differentiation, suggesting that *Map3k7* impacts an early lineage decision that directs cells to the SAN fate.

## Materials and methods

### Cell culture

CGR8 ES cells expressing the αMhc::*GFP* reporter [32] were obtained from Mark Mercola. R1 ES cells (obtained from ATCC) were transduced with the PGK::*Map3k7-*IRES*-GFP* expression virus and a second reporter αMhc::*mCherry*. These were maintained in ES cell growth medium. For differentiation studies, ES cells were passaged off MEFs and differentiated as EBs using the hanging drop method, as previously described [28].

### Construction of the *PGK*:: *Map3k7* expression vector

The open reading frame of mouse *Map3k7* was amplified by PCR from the pRK5m *-*WT*-*flag vector (a gift from Hiroshi Shibuya) and directionally cloned into the Sin18 pre.hPGK. IRES2.*eGFP*.PB vector (a gift from Mark Mercola), which drives expression of both the inserted gene and Green Fluorescent Protein (GFP) from the ubiquitous PGK promoter. Virus was produced using the second-generation lentiviral expression system [33].

### Real time PCR

EBs were collected on specific days of differentiation, RNA was isolated using Tri Reagent (Sigma), and cDNA was transcribed using Quantitect Reverse Transcription Kit (Qiagen). qRT-PCR reactions were carried out using 50 ng template/reaction in SybrGreen Master Mix (Roche), on a Roche LightCycler^®^ 480 Real-Time PCR Instrument, and analyzed with the LightCycler 480 software package (version 1.5.0.39). Crossing point data were first adjusted to reflect the efficiency of primer pairs by comparison to standard curves (based on dilution series over a total dynamic range of 1:1,000 or 1:10,000 for positive control cDNAs) and subsequently normalized to the ubiquitously expressed transcript *Gapdh*. Each data point represents averaged data from three technical replicates from a same time course experiment. Error bars represent standard error based on three technical replicates as calculated by Roche LightCycler software. A change in gene expression between *Map3k7*-overexpressing and wild-type EBs is considered relevant if the same change was observed in each of at least three biological replicates. Primers used in this study are listed in Table 1:

**Table 1:** Primer pairs used for real time PCR.

*Gene*	Forward primer	Reverse Primer
*T/bra*	AGCTTCGTGACGGCTGACAA	CGAGTCTGGGTGGATGTAG
*Fgf8*	GCTCATTGTGGAGACCGATAC	TTGCTCTTGGCAATTAGCTTC
*Gapdh*	AATGGATACGGCTACAGC	GTGCAGCGAACTTTATTG
*Hcn1*	TGCCAGTGTCCGAGCTGATA	TCTCTCGGTCATGCTTCACG
*Hcn2*	TGCTCAGCATGATCGTAGGC	CCCAAGGATGCTGTCCTCAT
*Hcn4*	ACCTGACGATGCTGTTGCTG	CTC TGC GGGTCAAGGATGAT
*Isl1*	GAGTCATCCGAGTGTGGTTTC	ACCATGGGAGTTCCTGTCATC
*Map3k7*	CGTAGATCCATCCAAGACTTGAC	GAGGTTGGTCCTGAGGTAGTGAT
*Map3k7* 3'UTR	CCAATGGCTCAGATAACTCCA	AACAAATGCAGCAAAGAGAGG
*Mhcα*	CATGCCAATGACGACCT	CCTACACTCCTGTACTGCC
*Mhcβ*	GGTGGCAAAGTCACTGCTGA	ACAGGCAGC CACTTGTAGGG
*Mlc2a*	CAGACCTGAAGGAGACCTATTCC	CTACCTCAGCAGGAGAGAACTTG
*Nkx2*.*5*	TTACCG GGAGCCTACGGTG	GCTTTCCGTCGCCGCCGTGCGCGTG
*Shox2*	TCCCCTGAACTGAAGGATCG	CAGTCGCTGGCTCAATTCCT
*Tbx3*	GTTTTGTCTGGGAGGGAGCA	CTTCAGCCCCGACTTCATA
*Tbx5*	CCAGCTCGGCGAAGGGATGTTT	CCGACGCCGTGTACCGAGTGAT
*cTnI*	CCGCCTCCAGAAAACTTCAG	CGTGAAGCTGTCGGCATAAG

### Immunocytochemistry

EBs were dissociated and cells were transferred to chamber slides. After attachment, cells were washed with PBS and fixed using 4% paraformaldehyde at room temperature (RT) for 15 minutes. Blocking was carried out for one hour using 3% fetal calf serum (FBS), 2% BSA and 0.5–1% Triton X-100 in PBS. Expression of cardiac contractile proteins was assessed using the anti-Sarcomeric Myosin (CT3) antibody (Developmental Studies Hybridoma Bank), goat polyclonal anti-*Troponin* (C-19) (Santa Cruz), anti-*Cx43* (Santa Cruz), anti-*CaV1*.*3* (NeuroMab), anti-*Kir3*.*1* (Santa Cruz) and anti-*Hcn4* (NeuroMab) antibodies. Cells were then stained with fluorescent-conjugated secondary antibodies, Alexa Fluor® 555 or Alexa Fluor® 488 (Invitrogen) diluted 1:1,000. The nucleus was visualized with DAPI and mounted in vectashield (Vector).

### Calcium imaging

Calcium imaging was performed on cardiomyocytes obtained from collagenase-dissociated EBs using the cell-permeant acetomethyl (AM) form of the calcium sensitive dye mag-Fluo-4 (Molecular Probes). Analysis was carried out 5–7 days after the initial dissociation of cells at day 16 of EB differentiation. The AM ester of the indicator dye was dissolved in 25μl of DMSO to a concentration of 2.45 mM. For cell loading, 12.5 μl of this solution was diluted into 1 mL of differentiation medium to a final concentration of 30.6 μM, and incubated for 30 minutes at RT. After initial incubation, cells were washed with differentiation medium for 30 minutes to remove unbound dye and allow complete de-esterification. Epifluorescence was recorded using one camera of an 80x80 pixel CardioCCD-sm Dual-Camera Imaging System (RedShirtImaging) at 1000Hz, digitized at 14 bits. Baseline recordings were performed on cells in differentiation medium at RT. Response to acetylcholine and norepinephrine was recorded at RT. Drug exposure was performed through a series of washes–three initial washes with differentiation medium, a drug wash with differentiation medium and drug, and a final wash with differentiation medium. After each wash, 10 s of epifluoresence was recorded at three time points, 15 s, 2 min, and 4 min. Data was stored and analyzed with the RedShirtImaging Cardioplex software.

### Growth curves

To determine if *Map3k7* impacts cell proliferation, the rate of growth of cells overexpressing *Map3k7* was compared to that of wild-type cells. *Map3k7*-overexpressing and wild-type ES cells were plated at identical densities and counted daily over the course of several days. The total cell number on each day was averaged over several trials. In parallel, these cells were differentiated as EBs and their growth rates were assessed during differentiation.

### Flow cytometry

Wild type R1 EBs and *Map3k7*-overexpressing EBs were collected and dissociated into single cell suspensions using 1 mg/ml Collagenase D in DMEM. Cells were washed with 1X PBS and fixed with 4% PFA at RT for 15 minutes. Permeabilization was carried out for 10 min in 1X PBS consisting of 0.5% BSA and 0.1% Triton X-100 (permeabilization buffer). After permeabilization, cells were incubated for 1 hour in permeabilization buffer containing mouse MF20 antibody (Developmental Studies Hybridoma Bank) diluted at 1:100. Compensation control samples were also prepared without the addition of MF20. Cells were then washed with permeabilization buffer and incubated for 1 hour with a FITC-conjugated goat polyclonal anti-mouse secondary antibody (abcam) diluted at 1:1000. After washing twice in permeabilization buffer, cells were resuspended in 1X PBS and filtered through an 80 μm sieve. Flow cytometry was performed with a Becton-Dickinson (B-D) FACScan, and data were acquired using the B-D CellQuest software. Dot plots were created with FSC-H and FL1-H on the x- and y-axes respectively, and FITC-treated negative controls (compensation controls) were used to eliminate non-cardiomyocytes during statistical analyses. Data is represented as fold difference compared to untreated wild-type EBs. Bars represent the average difference between wild-type and *Map3k7*-overexpressing cells over four separate trials. Error bars represent standard error. Statistical significance was determined by unpaired, two-tailed t-test.

### Electrophysiology

EB cells plated on gelatin-coated glass coverslips were placed in the experimental chamber (23°C), and superfused with Tyrode solution of the following composition (mM): 140 NaCl, 5.4 KCl, 2 CaCl_2_, 1 MgCl_2_, 5 HEPES, 10 glucose (pH 7.4). Membrane currents or action potentials (AP) from single cells or small clusters of cells were recorded using a computer equipped with pCLAMP 8, a Digidata 1322A series interface and Axopatch 1C amplifier (Molecular Devices). Only cells expressing mCherry fluorescence driven by the *MHCα* promoter were used for recording. The perforated patch clamp technique was employed. Borosilicate glass pipettes (Sutter Instrument) were filled with (mM) 130 aspartic acid, 146 KOH, 10 NaCl, 2 CaCl_2_, 5 EGTA, 10 HEPES, 2 Mg-ATP, 100 μg/ml amphotericin (pH 7.2). After forming a gigaseal, progress in electrical access was evaluated by monitoring capacitance currents induced by 20 ms pulses from -35 mV to -40 mV. AP and I_f_ were recorded when series resistance was reduced to 40–50 MΩ and 20–30 MΩ respectively. I_f_ was induced by voltage steps ranging from -35 to -125 mV with duration decrementing with more negative pulses, followed by a 5 s long pulse to -85 mV to measure tail current and 0.5 s deactivating pulse to -5 mV. Holding potential was -35 mV.

### Data curation

Raw data underlying figures that contain post-collection analysis by the investigators is provided as supporting data (S1 Tables).

## Results

### Overexpression of *Map3k7* by lentiviral vector

**Fig 1:**
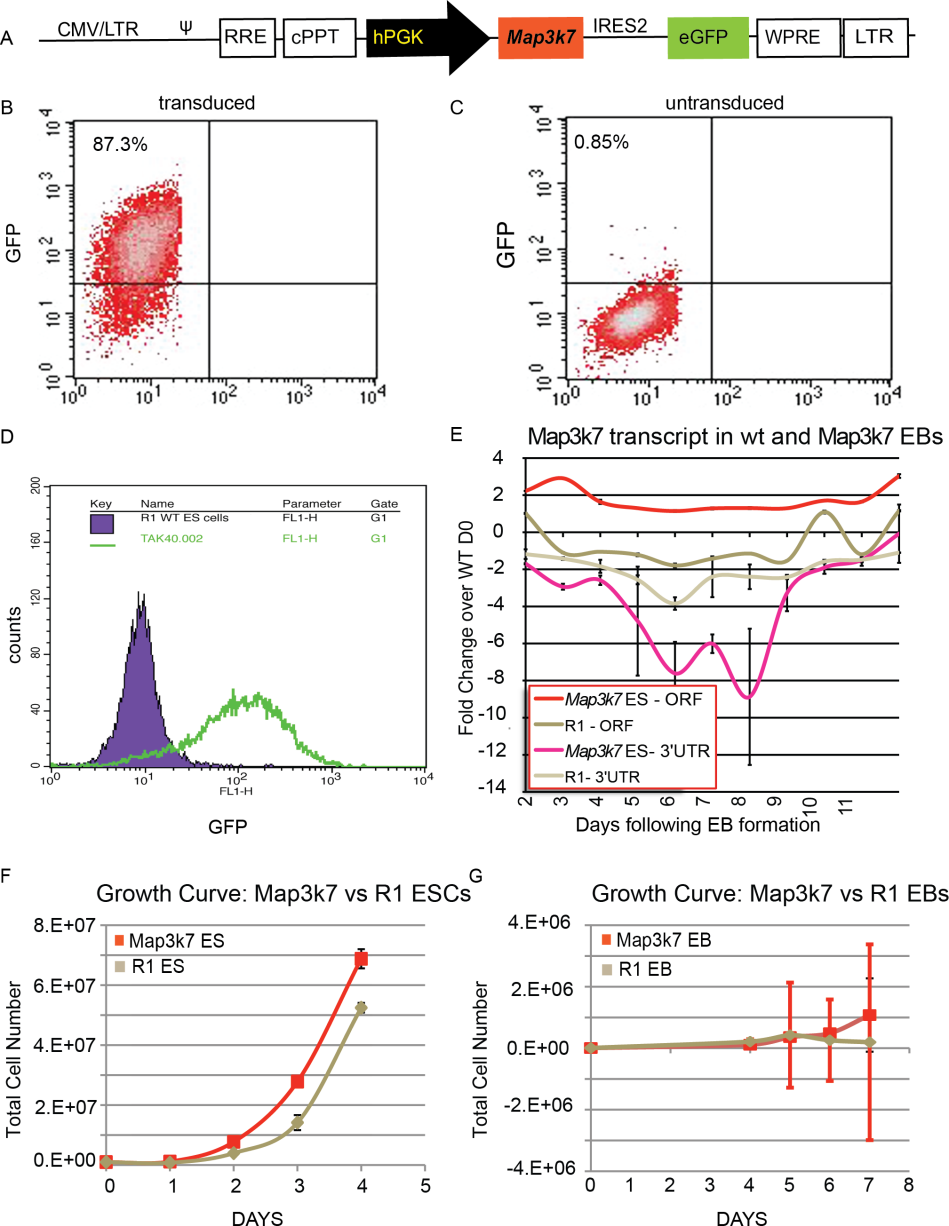
A. Schematic of lentiviral construct used to create an ES cell line stably overexpressing *Map3k7*. B-D. Flow cytometry analysis comparing green fluorescent protein (GFP) in *Map3k7*-overexpressing ES cells (B) to unmodified R1 cells (C). D. Cell count as compared to GFP fluorescence in untransduced (purple) and transduced (green) cells. E. qRT-PCR data showing overall *Map3k7* expression during EB differentiation (red line) as compared to unmodified ES cells. Error bars indicate standard deviation of three technical replicates from a single differentiation. F-G. Growth curves comparing rate of growth in *Map3k7*-overexpressing cells (red) as compared to unmodified R1 cells, when grown as ES cells (tan) (F) and during EB differentiation (G). Error bars indicate standard deviation across three biological replicates.

*Map3k7* was overexpressed in mouse R1 ES cells using a lentivirus driving *Map3k7* and green fluorescent protein (GFP) under the control of the strong ubiquitous human promoter, hPGK. The Sin18hPGK::*Map3k7*-IRES2-GFP (Fig 1A) virus was produced using the second-generation lentiviral expression system [33, 34], and ES cells were transduced using a high viral titer (multiplicity of infection (MOI) ranging from 25–40). Colonies expressing the transgene were identified by flow cytometry for high expression of GFP (Fig 1B–1D) and by qRT-PCR for the continuous overexpression of *Map3k7* mRNA during EB differentiation. EBs virally transduced with MOIs of 40 showed continuous two-fold overexpression of *Map3k7* mRNA as compared to unmodified R1 cells. Lower levels of infection resulted in either no increased expression, or decreases in the overall expression of *Map3k7*. Interestingly, endogenous *Map3k7* transcripts, assessed using primers specific to the *Map3k7* 3’ UTR, were dramatically downregulated during EB differentiation. This finding suggests that the *Map3k7* pathway is subject to an autoregulatory negative feedback loop that is only overcome by continuous overexpression of the gene under the PGK promoter (Fig 1E). *Map3k7*-transduced ES cells possessed a small but statistically significant growth rate advantage over wild-type ES cells, but there was no significant difference in the growth rates of *Map3k7* and wild-type EBs (1f, g).

### Cardiomyocytes derived from *Map3k7*-overexpressing ES cells are morphologically distinct from cardiac cells derived from wild-type ES cells

**Fig 2:**
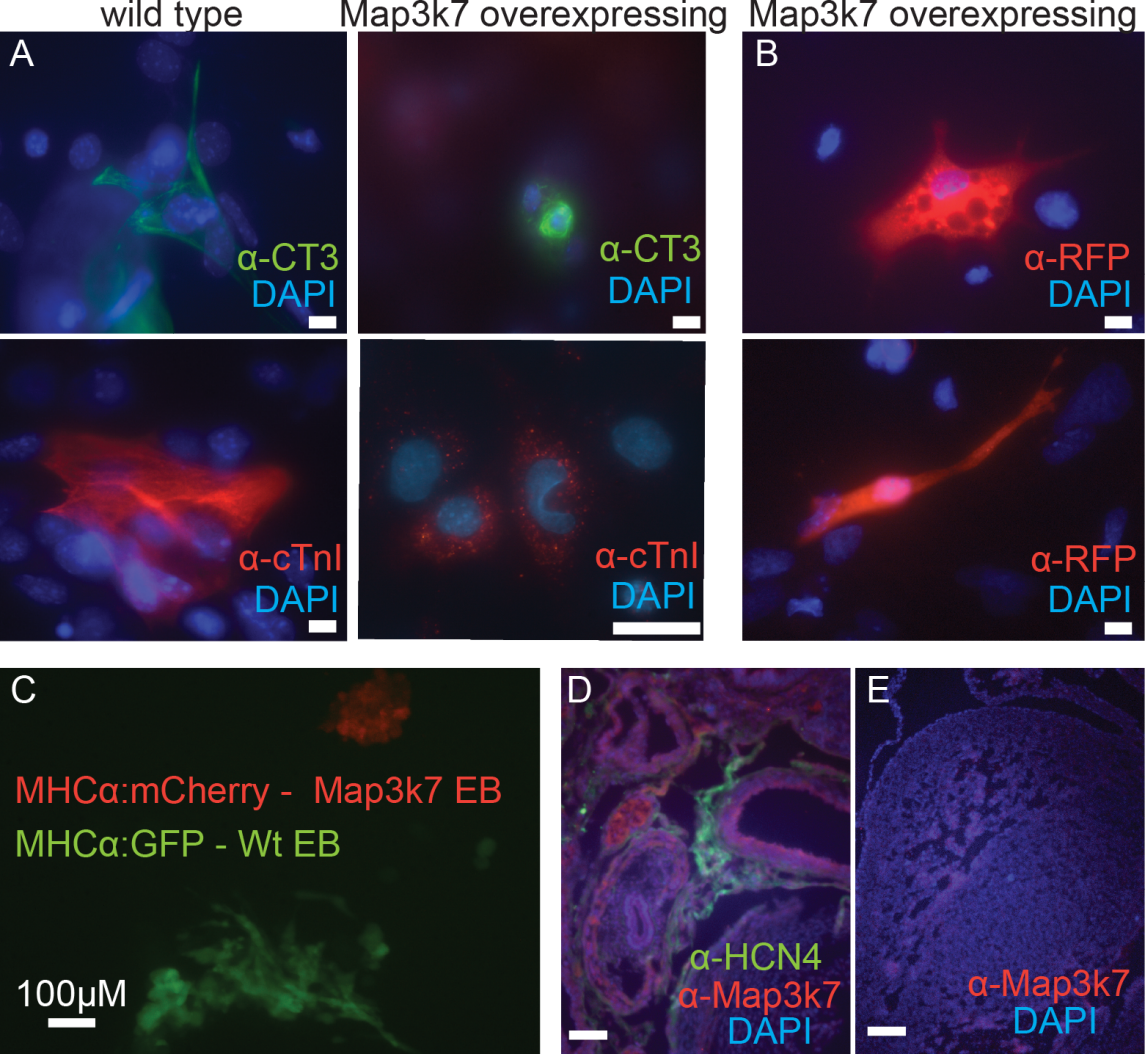
A. Immunocytochemistry showing that individual or small clusters of cardiomyocytes that form in wild-type and *Map3k7*-overexpressing EBs express both the MF20 epitope (anti-CT3 epitope, green) and *cardiac Troponin* (*cTnI*, red). Blue indicates DAPI signal in all panels. Note that Troponin protein is poorly organized in cardiac cells derived from *Map3k7*-overexpressing ES cells. Scale bars indicate 10 μM in panels stained for cTnI and 20μM in panels stained for MF20. B. After several weeks in culture *Map3k7*-overexpressing cells showed spider and spindle cell morphologies that are characteristic of mature SAN cells. C. Fluorescent image showing *Map3k7*-overexpressing cells that also possess the MHCα::mCherry reporter (marking cardiomyocytes with red fluorescence) and wild-type cells possessing the MHCα::GFP reporter (marking cardiomyocytes with green fluorescence). Note that cardiomyocytes derived from *Map3k7*-overexpressing cells possess a distinct morphology as compared to wild-type cardiomyocytes. Scale bar represents 100 μM. D. Anti- *Map3k7* antibody staining of 19 day old mouse embryo showing increased expression in the remnants of the sinus venous and overlapping with HCN4 positive cells. E. By contrast, low expression of *Map3k7* in the left ventricle of the same heart.

Both wild-type and *Map3k7*-overexpressing EBs produce cardiomyocytes recognized by cardiac specific antibodies against α-CT3 and α-cTnI (Fig 2A). There are several published protocols to enhance cardiomyocyte differentiation in EBs by addition of growth factors, however since *Map3k7* is known to interact with theses pathways we chose to differentiate them in the absence of growth factors. These protocols, while yielding an overall low yield of cardiomyocytes (1–4%) (not shown) allows us to specifically observe the effects of *Map3k7* without the confounding effects of growth factors that may effect its level of expression or phosphorylation state.

However, *Map3k7*-overexpressing cardiomyocytes were morphologically distinct from those differentiated from wild-type ES cells. The vast majority of *Map3k7*-overexpressing cardiomyocytes possessed a small round morphology that is characteristic of immature pacemakers (Fig 2A). At later time points some *Map3k7* overexpressing cardiomyoctyes adopted spider-shaped and spindle-shaped morphologies that are characteristic of mature SAN cells (Fig 2B) [35, 36]. These cardiomyocytes often showed poorly organized myofibrils, another hallmark of SAN cells [37] (Fig 2A). To further analyze this, clonal lines of *Map3k7*-overexpressing ES cells were established that also express the cardiac-specific fluorescent reporter *MHCα*::*mCherry* [32] (Fig 2C). It was noted that beating foci in *Map3k7*-overexpressing EBs (red cells in Fig 2C) had a dramatically different organization as compared to beating foci in wild-type EBs, which possess the *MHCα*::*GFP* reporter (green cells in Fig 2C). Wild type colonies were comprised of cardiomyocytes with large, well-organized myofibrils, whereas *Map3k7*-overexpressing colonies were organized in tight, rounded cell clusters.

### *Map3k7* is upregulated in the region of the sinus node and markedly downregulated in the ventricular myocardium

Cardiomyocytes derived from *Map3k7*-overexpressing EBs differentiate initially as small, round clusters whereas a previous study had demonstrated that *Map3k7* overexpression in the ventricular myocardium resulted in cardiac hypertrophy [38]. To understand how *Map3k7* overexpression might lead to such diametrically opposed outcomes in different developmental contexts, we examined *Map3k7* protein expression in embryonic mouse hearts.

In 14.5 day old mouse embryos, *Map3k7* protein (α-*Map3k7*) was expressed at comparatively higher levels in the atria and inflow tract region of the heart, including high expression in *Hcn4* positive cells of the SAN (Fig 2D). By contrast, *Map3k7* expression in the ventricular myocardium (Fig 1E) was very low or nearly absent, except in the trabeculae. This suggests that SAN cells normally express higher levels of *Map3k7* than ventricular cardiomyocytes.

### Cardiomyocytes derived from *Map3k7*-overexpressing EBs display physiological, electrophysiological, and molecular characteristics of the SAN

**Fig 3:**
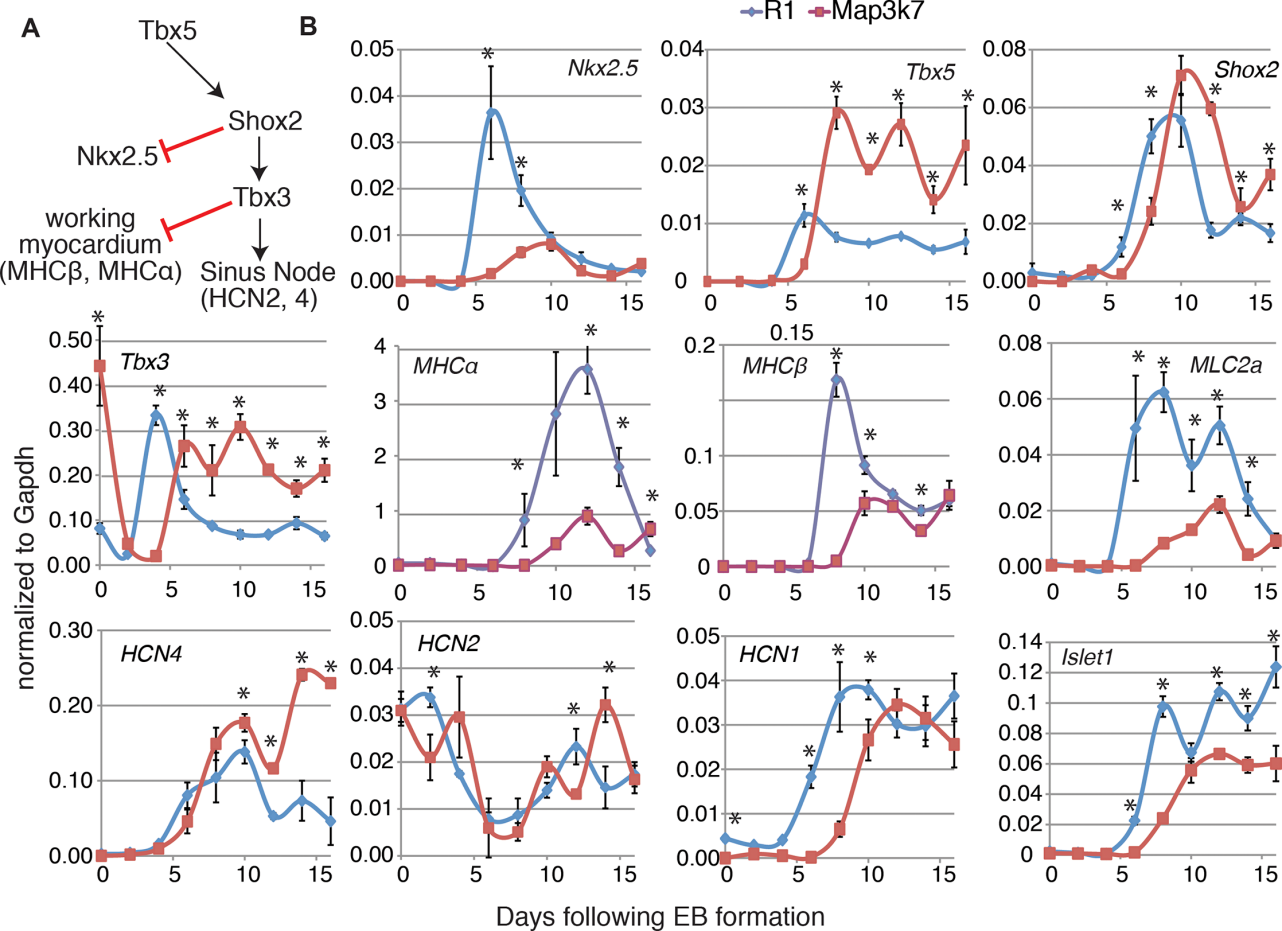
A. Diagram of the major transcriptional regulators of SAN differentiation in the mouse embryo. *Tbx5* activates *Shox2*, *Tbx3* and SAN-specific markers, including *Hcn2* and *Hcn4*. In addition, *Shox2* inhibits *Nkx2*.5 expression, and *Tbx3* inhibits markers for the working myocardium. B. qRT-PCR data showing changes in the expression or timing of SAN related genes. Blue line indicates relative gene expression normalized to expression of *Gapdh* over time in wild-type R1 EBs and red lines indicate gene expression in *Map3k7*-overexpressing EBs. Error bars indicate standard error from three technical replicates. Upregulation or downregulation of each gene was considered relevant only if relative expression trends were the same in each of a minimum of three biological replicates. Error bars represent standard error from three technical replicates. Statistical significance was determined by t-test. (*) represent p<0.006.

To determine if *Map3k7* influences the differentiation of cardiomyocytes to the SAN fate, we used qRT-PCR to examine markers that are known to influence the SAN fate *in vivo* (reviewed in [12])) (Fig 3A). *Map3k7*-overexpressing ES cells were differentiated as EBs and assessed by qRT-PCR for cardiac marker expression over 16 days of differentiation (Fig 3B). During EB differentiation, continuous overexpression of *Map3k7* had a profound impact on the transcriptional expression of the cardiac progenitor markers *Nkx2*.*5* and *Tbx5*. By day 5 of differentiation, *Nkx2*.*5* transcription was decreased in *Map3k7* cells and by day 7, *Tbx5* was dramatically increased in *Map3k7*-overexpressing EBs.

Within approximately the same differentiation window, mRNAs encoding the SAN-specific transcription factors *Shox2* and *Tbx3* were upregulated and cardiac contractile proteins, *Mhcα*, *Mhcβ*, *Mlc2a*, were markedly decreased. Other SAN-specific markers were either unchanged as compared to wild-type EBs (*Hcn2*) or upregulated (*Hcn4*). *Hcn1*, which is not expressed in the SAN of the mouse embryo was expressed at similar levels as compared to wild-type EBs, but the timing of expression was different.

*Isl1* is expressed in cardiac precursors and is down regulated in most differentiated myocardial cells. However, sinus node cells continue to express *Isl1* [39]. In wild-type EBs, we typically observe three pulses of *Isl1* expression. While one of these pulses was normal in *Map3k7*-overexpressing cells, the other two were decreased; however, the overall expression was similar in wild-type and *Map3k7* expressing cells. Since *Isl1* is non-specific for the node, we examined another marker of SAN precursor, *Tbx18*. As with *Isl1*, *Tbx18* (data not shown) was not significantly different between the two populations. This data suggests that *Map3k7* acts on established SAN precursors but does not determine the size of the precursor population.

To address this, we examined the early mesoderm markers *T/brachyury* and *Fgf8*, and the cardiac progenitor marker *Mesp1*, none of which were affected by *Map3k7* overexpression (data not shown). Together, these data support the hypothesis that *Map3k7* influences lineage specialization within the heart rather than progenitor cell proliferation.

**Fig 4:**
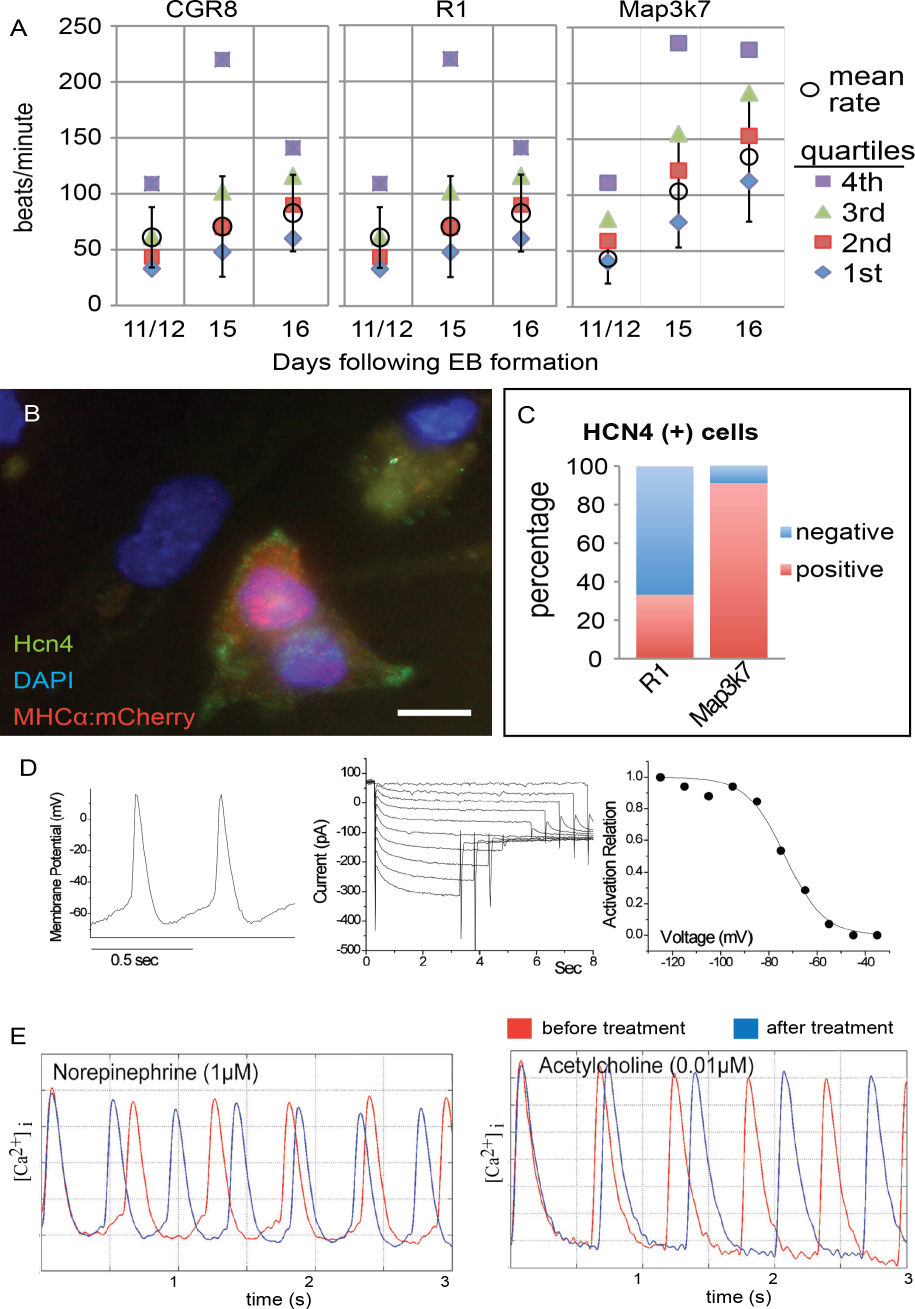
A. Beat Rate Data showing quartile beat rate data for 100 beating foci for two wild type mouse strains and *Map3k7* overexpressing cells. B. Immunocytochemistry showing overlap of the cardiac reporter with the SAN specific marker *Hcn4*. C. Summary of this data showing the % of Hcn4 expressing cardiomyocytes is dramatically increased in the *Map3k7*-overexpressing cells. D. Perforated patch recording of automaticity and pacemaker current in mCherry expressing cardiomyocytes derived from *Map3k7*-overexpressing ES cells. Left: Spontaneous APs. Middle: Pacemaker current recorded from the same cell. Right: calculated activation relation from the same cell, showing current activation within the diastolic potential range. E. Calcium transients in cardiomyocytes derived from *Map3k7*-overexpressing ES cells before (red trace) and after (blue trace) treatment with either 1μM norepinephrine or 0.01μM acetylcholine, as indicated. Beating in these cardiomyocytes accelerates in response to norepinephrine and slows down in response to acetylcholine.

Beat rates for individual contracting foci were scored on days 11 and 12, and then on days 15 and 16 of EB differentiation (Fig 4). On days 11 and 12, EBs from each of the three cell lines had beating areas contracting at approximately 50 beats per minute (bpm). By days 15/16 the rate of beating in wild-type EBs had increased to 60–70 bpm. By contrast, *Map3k7*-overexpressing EBs had an average beat rate greater than 100 bpm at room temperature. Of the beating loci quantified on days 15 and 16, 12/42 (29%) of R1 EBs, 13/32 (40%) of CGR8 EBs and 46/59 (78%) of *Map3k7* EBs had areas beating above 90 bpm. Both SAN and atrial cells beat more rapidly in culture than ventricular cardiomyocytes [18]. To distinguish between these possibilities, we examined by immunocytochemistry the expression of Hcn4, which is expressed in the SAN but not in atrial cells, and found that it was highly expressed in cardiomyocytes derived from *Map3k7* EBs (Fig 4B), but was rarely observed in wild-type EBs. To quantify this, individual or small clusters of cardiomyocytes (as determined by the expression of the mCherry reporter) were scored for expression of HCN4. Approximately one third of all wild-type cardiomyocytes expressed Hcn4. In contrast, nearly all *Map3k7*-overexpressing cardiomyocytes expressed *Hcn4* (Fig 4C).

We performed perforated patch clamp studies on mCherry-expressing cardiomyocytes to test if *Map3k7*-overexpressing cardiomyocytes have physiological and electrophysiological characteristics of the SAN. Our initial patch clamp studies on cardiac cells derived from *Map3k7*-overexpressing cell lines demonstrated that most of the cells tested had action potential morphologies characterized by a phase 4 (diastolic) depolarization and/or expressed the I_f_ current, suggesting that these cardiac cells were either primary pacemaker or mature SAN cells. However, when these cells were treated with a low concentration of norepinephrine (0.01μM) to ensure continual activation of the *Map3k7* signaling pathway [40], 100% (6/6) of the patched cells exhibited action potentials identical to those of isolated mouse sinoatrial cells [41] and 4 of 4 showed physiologically appropriate expression of the I_f_ current activating over a physiologically appropriate voltage range (Fig 4D).

While many cardiac cells adjust their rate of beating in response to β-adrenergic and cholinergic stimulation, SAN cells are required do so. To test if the cardiomyocytes isolated from *Map3k7*-overexpressing EBs behave physiologically like mouse SAN cells, calcium transient data was measured in mCherry-expressing (cardiac) cells derived from *Map3k7*-overexpressing EBs using a calcium sensitive dye. Consistent with a SAN identity, cells exposed to a relatively high dosage of norepinephrine (1 μM) showed an increased calcium transient rate in all cardiomyocytes tested, with an average increase of 36% (p<0.0006), whereas addition of 0.01 μM acetylcholine decreased the calcium transient rate by an average of 7.5% in 5 of 6 (83%) cardiomyocytes (p<0.0006) (Fig 4E). Notably, the dose of norepinephrine that was required to speed up the rate of calcium transients was approximately 100-fold greater than the dose used to stimulate electrophysiological differentiation. These data suggest that Map3k7 overexpression plays a role in the electrophysiological maturation of SAN cells which is separate from its ability to change beat rate in response to beta-adrenergic or cholinergic stimulation.

**Fig 5:**
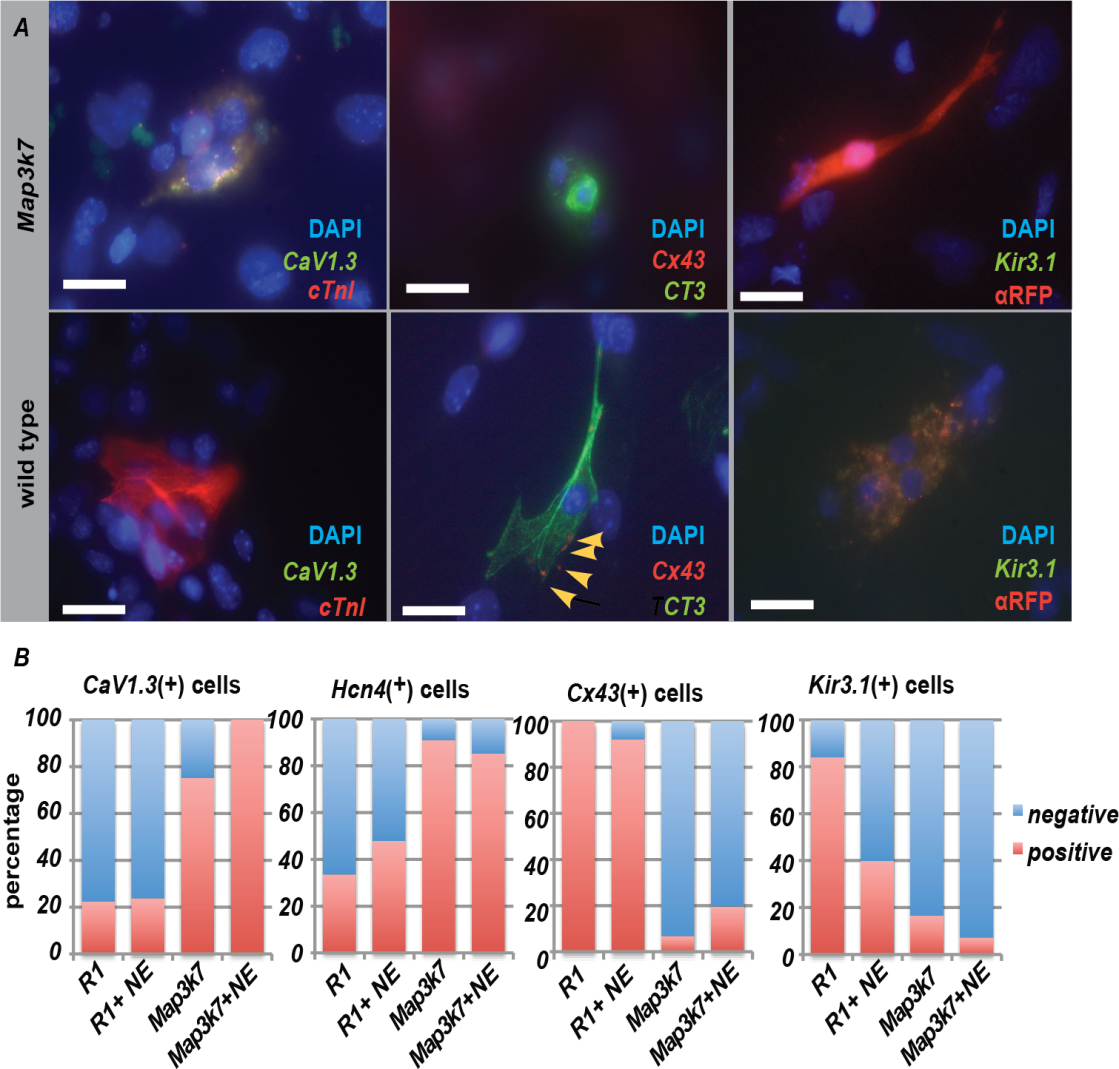
Immunocytochemistry on individual or small clusters of wild type or *Map3k7* overexpressing cardiomyocytes (as indicated by expression of cTnI, red, in CaV1.3 and *Kir3*.*1* panels) or CT3 monoclonal antibody (green, in Cx43 panels) indicating that the SAN-specific calcium channel *CaV1*.*3* (green) is expressed in *Map3k7* cardiomyocytes but not in most cardiac cells derived from wild type ES cells. By contrast Cx43 (red and red arrow heads) and *Kir3*.*1* (green) are expressed in most wild type cardiomyocytes but not in cardiac cells derived from *Map3k7* overexpressing EBs. Blue indicates DAPI staining in all panels. Scale bars in all figures represent 20μM.

To determine if low doses of norephinephrine effects the expression of channel protein, we analyzed the expression of several ion channel and gap junction proteins by immunocytochemistry (Fig 5A). These included *Kir3*.*1*, *Connexin43 (Cx43)*, and *CaV1*.*3*, all of which are differentially expressed between SAN and the working myocardium [15, 42]. Individual or small clusters of cardiomyocytes (as determined by the expression of the mCherry reporter) were scored for expression of these markers (Fig 5B). The percentage of cells with expression was compared between cardiomyocytes derived from unmodified R1 ES cells and those that were stabily transduced with the PGK::*Map3k7* overexpression vector. These were also compared to *Map3k7* overexpressing cells that were treated with a low dose (0.01μM) norepinephrine. Both *Hcn4* (Figs 2C and 5B) and the calcium channel *CaV1*.*3* were detected in approximately 20% of cardiomyocytes of wild-type origin. However, more than 80% of cardiomyocytes derived from *Map3k7*-overexpressing EBs expressed these markers. Addition of low dose norepinephrine did not have a major impact on either *Hcn4* or *CaV1*.*3* expression levels. In contrast, Cx43 and Kir3.1 were expressed in most wild-type cardiomyocytes but only in less than 20% of of *Map3k7*-overexpressing cardiac cells. In addition, the expression of *Kir3*.*1* was dramatically decreased in wild-type cardiomyocytes after the addition of low dose norepinephrine. Therefore, these data not only support a SAN-like identity for *Map3k7*-overexpressing cardiomyocytes, but also suggest that specific channel proteins might be affected by the continuous activation of this pathway by norepinephrine.

## Discussion

### Lineage-specific differentiation of sinoatrial node cells *in vitro*

Here we describe a novel protocol that directs the differentiation of ES-derived myocardial cells toward the SAN sublineage.These cells upregulate the transcriptional network that mediates SAN differentiation *in vivo*, express markers for the SAN and display physiologic and electrophysiological characteristics of the node. Action potential morphologies, the mis-alignment of contractile fibers, rapid beat rate and the severe down-regulation of markers for the working myocardium eliminate the possibility that these cells represent ventricular myocardium. In addition, while both SAN and atrial cells beat fast, *Map3k7*-overexpressing cells express *Hcn4*, which is not expressed in the atrium. Finally while both SAN and Purkinje fibers express the I_f_ current, Purkinje cells do not respond to the addition of acetylcholine unless the rate has already been accelerated by catecholamine (accentuated antagonism), whereas SAN cells do [43], eliminating the possibility that these are Purkinje-like. In short, these cells can only be SAN.

### When does *Map3k7* function in SAN differentiation?

In these studies, we overexpressed *Map3k7* using the ubiquitously expressed human PGK promoter and because of this, *Map3k7* could function at any stage of EB differentiation to direct SAN differentiation. Cardiac cells in these cultures begin to beat more rapidly than wild-type cells between days 12 and 15 of differentiation. However, it is unclear if this sudden increase in beating represents a change of fate or simply reflects the maturation of cells already fated to become SAN. In the mouse embryo, cells fated to give rise to the SAN can be distinguished as early as 8.5 dpc as a region in the atrial wall that co-expresses *Tbx18* and *Isl1*, but which lacks expression of *Nkx2*.*5*[44]. These data suggest that the SAN fate separates from other myocardial lineages quite early in mouse development. Marker analysis of *Map3k7*-overexpressing EBs supports this idea. When cardiac markers were assessed over time in differentiating EBs, differences were observed as early as day 6. More specifically, we observed an increase in *Tbx5* and a decrease in *Nkx2*.*5*. At the same time, there was a marked decrease in the activation of contractile proteins such as *Mlc2a*, *Mhcβ* and *Mhcα*. These data demonstrate that *Map3k7* mediates an early lineage decision biasing myocardial differentiation away from atrial and ventricular fates and toward the SAN fate.

Alternatively, it is possible that *Map3k7* overexpression simply causes cells to beat more rapidly than wild-type cardiomyocytes, which as a consequence, causes electrophysiological remodeling of cardiomyocytes to a SAN-like phenotype. This is, however, unlikely for several reasons. First, changes in gene expression occur prior to the onset of beating, suggesting that rapid beating is a consequence of channel maturation within cells already fated to become SAN and not vice versa. Also, previous studies, in which *Map3k7* was overexpressed in ventricular myocardial cells, did not result in remodeling of those cells to a SAN-like morphology [31] but rather caused these cells to become hypertrophic and ultimately to fail.

A transcriptional network mediating SAN differentiation in the mouse has been established by genetic studies. In these studies it appears that *Tbx5* expression in the area of the sinus venosus activates expression of *Shox2*, which in turn, both inhibits expression of *Nkx2*.*5* and activates expression of the transcriptional repressor, *Tbx3* [7, 8]. Our studies place *Map3k7* activity upstream of this pathway, since all of these factors were profoundly impacted by *Map3k7* overexpression.

On the other hand, cells fated to become SAN in mouse can be distinguished early in development based on co-expression of *Tbx18* and *Isl1*[44]. In addition, *Isl1*, while initially expressed in in all cardiac precursors, is down regulated in all differentiated myocardial cells except those in the sinus node[39]. Overexpression of *Map3k7* did not impact the expression of *Tbx18* and had a complicated effect on the timing but not overall expression of *Isl1*. This suggests that these factors are either upstream of *Map3k7* or in a parallel pathway.

### The implications for regenerative medicine

Protocols for lineage specific differentiation of cardiomyocytes have tremendous potential for regenerative medicine. First, they will provide the tools required for screens that will eventually identify novel markers of the SAN fate. These screens could be comprised of either microarray or proteomic analyses or could involve comparing epigenetic markers to identify enhancers that are activated at different stages of SAN differentiation [45].

Second, these cells could serve as the basis for pharmacological screens on pacemaker cells derived from human ES cells or from patient-specific iPS cells. This will potentially allow for the identification of factors with specific therapeutic benefit to, or toxic effects on, the SAN.

Finally, these cells could serve as the basis for the development of biological pacemakers. Unlike skeletal muscle, cardiomyocytes in adult mammals have little or no ability to regenerate after injury. Instead, damage results in the formation of apoptotic and necrotic cells that are eventually replaced by fibroblasts and scar tissue. In particular, damage to the heart’s pacemaker, the SAN, results in bradycardia, arrhythmia, and ultimately, heart failure. Because of the central importance of the SAN to cardiac function, in addition to the limitations of mechanical pacemakers, a number of proposals have been put forward for the creation of biological pacemakers and several proof-of-principle experiments have demonstrated the potential efficacy of this approach. First, non-pacemaker cardiac cells could be converted to pacemaker function by vector-mediated introduction of factors that convey pacemaker function. For example, the viral introduction of *Hcn2* proteins into the left branch bundle of dogs in complete AV block, increased the basal rate of beating, decreased the dependence on mechanical pacemakers [46] and increased the heart’s responsiveness to exogenous adrenergic stimulation and emotional arousal [46, 47]. Similarly, single ventricular myocytes derived from rats or guinea pigs or human mesenchymal stem cells that are virally transduced with Hcn family members, adopted electrophysiological characteristics of the SAN [48–50]. Other types of genetic manipulations, such as introducing constructs that increase cAMP production [51], or which suppress the *Kir2*.*1* channel[52], have also been used to activate spontaneous contractions and other pacemaker-like activities in adult cardiac cells that do not normally beat spontaneously. A potential problem with this approach is that adoption of fully functional pacemaker activity in non-SAN cells will likely depend on complex interactions between cell surface channels and proteins regulating calcium concentration in the sarcoplasmic reticulum (reviewed in [53]), and may therefore require the simultaneous activation and/or deactivation of multiple genes.

Another approach that is currently being tested is the introduction of xenografts of pacemaker-like cells into the hearts of SAN-damaged animals. Indeed, both implanted EBs and ES-derived cardiomyocytes [54, 55], in addition to the Hcn-modified cells described above [49, 50], have been tried in animal models and have shown to transiently engraft into host tissue and/or activate spontaneous action potentials. Nevertheless, it should be noted that most ES-derived cardiomyocytes display spontaneous beating early in their development, but this native pacemaker activity is lost as they mature into working myocardium. For example, the spontaneously contracting cells engrafted by Kehat expressed the gap junction protein *Cx43*, which is not expressed in sinoatrial node cells, suggesting that these were immature ventricular or atrial cardiomyocytes rather than SAN cells. In other words, for this approach to work, it is likely that a purified population of true SAN cells will have to be identified. As a proof of principle, cells enzymatically isolated from the sinoatrial nodes of canines, that were then re-implanted as grafts into the apex of the ventricle, were also effective in initiating action potentials and temporarily reducing the dependence on electronic pacemakers [56]. The *Map3k7* overexpression protocol described here may therefore be ideal for the production of medically applicable sinoatrial node cells because it appears to specifically select SAN differentiation at the expense of other myocardial cell types, and thus will allow for an expandable source of potential donor cells for engraftment.

## Supporting information

S1 Tables Original data underlying Figures: Four Excel sheets including: 1) growth rate data 2) beat rates for individual embryos bodies 3) Calcium transient rate data in response to Norepinephrine 4) Calcium transient rate data in response to Norepinephrine.
(XLS)
